# Navigating Mental Health Support in Tunisia’s Digital Age: Preferences, Challenges, and Paradoxes-An online survey

**DOI:** 10.1192/j.eurpsy.2024.1136

**Published:** 2024-08-27

**Authors:** O. Chhaider, M. Lagha, A. Adouni, I. Ben Romdhane, W. Homri, R. Labbane

**Affiliations:** ^1^Psychiatry C; ^2^Razi hospital, Manouba, Tunisia

## Abstract

**Introduction:**

The digital age has transformed mental health support in Tunisia. This study explores how individuals seek assistance for mental health concerns online, considering their comfort levels, preferences, and decision-making factors.

**Objectives:**

This study aims to: Investigate the comfort levels of Tunisians when discussing mental health concerns online Identify preferred online sources for mental health support Explore factors influencing the choice between online sources and mental health professionals

**Methods:**

To unearth these insights, an exhaustive online survey was meticulously conducted. This survey was posted online on different social media platforms and cast a wide net, drawing responses from an eclectic cross-section of Tunisian society. The survey methodically gathered data on participants’ demographics, their inclinations towards online avenues for seeking assistance, and the multifaceted factors that sway their choices in this digital age.

**Results:**

Intriguingly, the results of this study illuminate several key findings:

**Comfort Levels:** A striking 47% of respondents expressed their unease about discussing their mental health concerns online. This statistic vividly underscores the intricate interplay between digital platforms and the persistent social stigma surrounding mental health issues.

**Preferences for Online Sources:** The study notably revealed that mental health apps and online counseling websites are emerging as the favored choices among those seeking support. This underscores the surging significance of digital mental health solutions tailored to individual needs.

**Factors Influencing Preferences:** An array of factors sways the preference for online sources. Among them, the allure of anonymity, the appeal of convenience, and the perception of limited access to in-person mental health professionals were prevalent. Additionally, financial constraints emerged as a notable consideration in the decision-making process.

**Trust in Online Information:** In contrast, individuals who leaned toward seeking assistance from mental health professionals stressed the pivotal importance of professional expertise, personalized guidance, and a comprehensive understanding of their concerns. Importantly, 38% expressed reservations about the reliability of online mental health information, underscoring the critical role of trust in the process.

**Conclusions:**

This study sheds light on the nuanced process of seeking mental health assistance in the digital age. It emphasizes the need to address mental health stigma and improve online resource credibility. The findings highlight the importance of comprehensive mental health strategies that integrate digital solutions and traditional professional care, catering to diverse preferences and needs in Tunisia.

**Disclosure of Interest:**

None Declared

